# Mutation in the Endo-β-1,4-glucanase (KORRIGAN) Is Responsible for Thick Leaf Phenotype in Sorghum

**DOI:** 10.3390/plants11243531

**Published:** 2022-12-15

**Authors:** Lavanya Mendu, Gayani Jalathge, Kamalpreet Kaur Dhillon, Nagendra Pratap Singh, Vimal Kumar Balasubramanian, Rebecca Fewou, Dennis C. Gitz, Junping Chen, Zhanguo Xin, Venugopal Mendu

**Affiliations:** 1 Department of Plant Sciences and Plant Pathology, Montana State University, Bozeman, MT 59717, USA; 2 Department of Plant and Soil Science, Texas Tech University, Lubbock, TX 79409, USA; 3 Faculty of Science, University of Angers, 49000 Angers, France; 4 U. S. Department of Agriculture, Agriculture Research Service, Lubbock, TX 79415, USA

**Keywords:** sorghum, cell wall, endo-1,4-β-glucanase, KORRIGAN, *thick leaf* (*thl*), SbKORRIGAN, biofuel

## Abstract

Sorghum [*Sorghum bicolor* (L.) Moench] is an important crop for food, feed, and fuel production. Particularly, sorghum is targeted for cellulosic ethanol production. Extraction of cellulose from cell walls is a key process in cellulosic ethanol production, and understanding the components involved in cellulose synthesis is important for both fundamental and applied research. Despite the significance in the biofuel industry, the genes involved in sorghum cell wall biosynthesis, modification, and degradation have not been characterized. In this study, we have identified and characterized three allelic thick leaf mutants (*thl1*, *thl2*, and *thl3*). Bulked Segregant Analysis sequencing (BSAseq) showed that the causal mutation for the *thl* phenotype is in endo-1,4-β-glucanase gene (*SbKOR1*). Consistent with the causal gene function, the *thl* mutants showed decreased crystalline cellulose content in the stem tissues. The *SbKOR1* function was characterized using Arabidopsis endo-1,4-β-glucanase gene mutant (*rsw2-1*). Complementation of Arabidopsis with *SbKOR1* (native Arabidopsis promoter and overexpression by 35S promoter) restored the radial swelling phenotype of *rsw2-1* mutant, proving that *SbKOR1* functions as endo-1,4-β-glucanase. Overall, the present study has identified and characterized sorghum endo-1,4-β-glucanase gene function, laying the foundation for future research on cell wall biosynthesis and engineering of sorghum for biofuel production.

## 1. Introduction

The cell wall plays a significant role in plant growth and development by maintaining turgor pressure, defining cell shape, and maintaining rigidity [1,2,3,4]. In addition, plant cell walls protect the plants from biotic and abiotic stresses. Cell walls are composed of several complex matrix polysaccharides in which cellulose is the main load bearing component [3,4]. Cellulose is a homopolymer of β-1,4 linked glucose (Glc) with disaccharide cellobiose repeats as structural units [4,5]. The inter- and intra-molecular hydrogen bonding of glucose chains forms the crystalline structure of the cellulose [4,6,7]. Cellulose is synthesized by plasma membrane embedded Cellulose Synthase Complexes (CSC) utilizing glucose molecules as substrates [4,8]. Cellulose synthases belong to a large family of glycosyltransferases family 2 (GT2) [4,7,9,10]. The CSC was first pictured as a hexametric rosette structure in higher plants using a Freeze-fracture transmission electron microscopy, which are secreted to the plasma membrane by Golgi complex [11,12,13]. However, the most recent model suggests that there are three distinct isoforms of CESA necessary for CSC assembly and function [5,9,14,15]. Both genetic and biochemical evidence showed that different CESA isoforms exist and assemble into separate CSCs for primary and secondary cell wall cellulose biosynthesis. In *Arabidopsis thaliana*, CESA1, CESA3, and CESA6 are necessary for primary cell wall cellulose biosynthesis, while CESA4, CESA7, and CESA8 are required for the secondary cell wall cellulose biosynthesis [16,17,18,19,20]. 

KORRIGAN (KOR), a member of the 1,4-β-D glucanase family that hydrolyzes beta 1,4-D glucan chains and takes part in plant cell wall modifications [2,21,22]. *KOR* function was first identified in *Arabidopsis* in a dwarf T-DNA insertion mutant (*kor1-1*) in the promoter region, and mutant showed reduced hypocotyl length due to low levels of *KOR* mRNA and protein content, suggesting its role in cell elongation [2,23]. Another stronger KORRIGAN, allele (*kor1-2*) resulted in much dwarfed phenotype with multinucleate cells, aberrant cell plates and incomplete cell walls, and abnormal seeds with a key role in cytokinesis [2]. KOR contains an N-terminal membrane anchor region and cytosolic domain [21] and was found to be a part of the CSC [24]. Analysis of Arabidopsis *KOR* mutants showed changes in the cellulose content both in the primary and secondary cell walls. Three *KOR* genes (*KOR1*, *KOR2*, and *KOR3*) were identified and characterized in *Arabidopsis* and are suggested to be necessary for cellulose synthesis by acting as a proofreader in the assembly of glucose in cellulose microfibrils [25,26]. *KOR1* is the predominant cellulase present throughout the plant where *KOR2* and *KOR3* gene functions were restricted to specific cell types in Arabidopsis [27]. Arabidopsis *KOR1* mutant alleles *kor1-1*, *acw-1*, *and irx2*, show defects in cellulose biosynthesis and in abnormal primary and secondary cell wall cellulose biosynthesis [25]. The *Root swelling 2* (*RSW2*) alleles (*rsw2-1* to *4*) were shown to be allelic to *KORRIGAN* [21] and were identified as conditional mutants involved in cell wall biosynthesis [23,28]. These mutants show normal root phenotype at 18 °C but display radial swelling of root tip when transferred to 31 °C. At the non-permissive temperature of 31 °C, the *rsw2*/*acw1* mutant showed a reduced activity of KOR, resulting in 40% less cellulose than the wild type, revealing the role of KOR in cellulose biosynthesis [29]. 

The present study identified and characterized endo-1,4-β-glucanase mutants in sorghum. Previously, we have isolated sorghum thick leaf (*thl*) mutants and described the phenotypes [30]; however, the causal mutation for the phenotype is not known. The present study identified the causal mutations using the BSAseq (Bulked Segregant Analysis sequencing) approach using whole genome re-sequencing. The results showed that mutation in *SbKOR1* gene is responsible for the three thick leaf mutant phenotypes (*thl1*, *thl2,* and *thl3*). To further confirm the role of *SbKOR1* in cellulose synthesis, we have isolated *SbKOR1* gene and complemented Arabidopsis *rsw2-1* mutants. Results established that *SbKOR1* was able to complement *rsw2-1* mutant phenotype. Overall, our study identified the causal mutations of *thl* mutants and confirmed the role of *SbKOR* gene using the Arabidopsis model system. The present study is the first study identifying endo-1,4-β-glucanase mutant from monocot species.

## 2. Results

### 2.1. Identification and Characterization of thl Mutants

**Figure 1:**
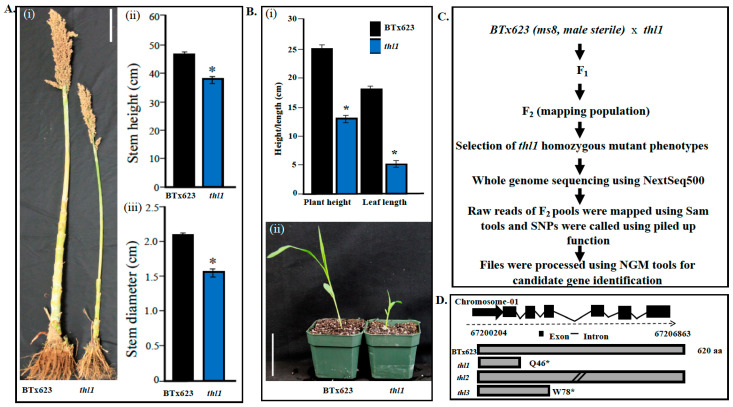
Phenotyping and mapping of sorghum *thl1* mutant locus using MutMap approach. (**A**). (i) Comparison of BTx623 and *thl1* mutants. (ii) Graph showing stem height of BTx623 and *thl1*. Scale bar: 10 cm. (iii) Graph showing stem diameter of BTx623 and *thl1*. (**B**). (i) Graph showing seedling height and leaf length of BTx623 and *thl1*. (ii) Images of BTx623 and *thl1* seedlings. Scale bar: 9 cm. (**C**). Flow chart showing generation of mapping population and *thl1* mutant mapping using MutMap approach. (**D**). Gene structure of *THL1* (exons and introns) and protein length of BTx623 and *thl* mutants. * Represents significant difference.

Genetic variation generated by natural or induced mutation is the source of novel traits for crop improvement. Ethyl Methane Sulfonate (EMS) produces random mutations resulting in novel traits. Sorghum mutant population was generated using EMS mutagenesis, and several mutants were characterized [31], including thick leaf mutant (*thl*) [30]. The present study identified and characterized three *thick leaf* mutants (*thl1*, *thl2*, *thl3*). The *thl* mutants (*thl1*, *thl2*, and *thl3*) along with un mutated BTx623 were grown in the field for phenotyping for plant height and stem diameter. Statistical analysis showed a significant reduction in plant height and stem diameter of *thl1* mutant (at the 6th node) compared to BTx623 (Figure 1A). Consistent with the field data, seedling phenotyping data also showed a significant reduction in overall growth (height), stem diameter, and leaf length in *thl1* mutant compared to BTx623 (Figure 1B).

Mapping populations were generated to identify the causal mutation using whole genome resequencing and bulked segregant analysis (Figure 1C). The *thl1* mutants were crossed with *ms8*, a male sterile isogenic line of BTx623 [32,33] to produce F_1_ seeds [31]. The F_1_ seeds were germinated to produce F_2_ mapping population. Then, homozygous *thl1* plants were identified visually by thick leaf phenotype and genomic DNA was extracted. The pooled genomic DNA was sequenced using the NextGen sequencing and BSAseq approach, as described [34]. In silico analysis identified the candidate mutant gene as *Sb01g036480* on chromosome 1. Nucleotide and protein sequence blast results showed that the *Sb01g036480* is a homolog of the Arabidopsis *KORRIGAN* gene. The mutation caused a truncated protein due to premature stop codon at amino acid position 46 (Q46*) in *thl1* mutant (Figure 1D) (Supplementary Figures S1 and S2). Sequencing of mapping populations and analysis of *thl2* and *thl3* also mapped the causal mutation to *Sb01g036480* (named as *SbKOR1* from here onwards). The *thl2* phenotype is due to a mutation in the splice site donor mutation, and *thl3* phenotype is due to a premature stop codon (W78*), resulting in truncated protein (Supplementary Table S1).

### 2.2. Phylogenetic Analysis Showed a Highly Conserved Nature of the KORRIGAN Proteins

**Figure 2:**
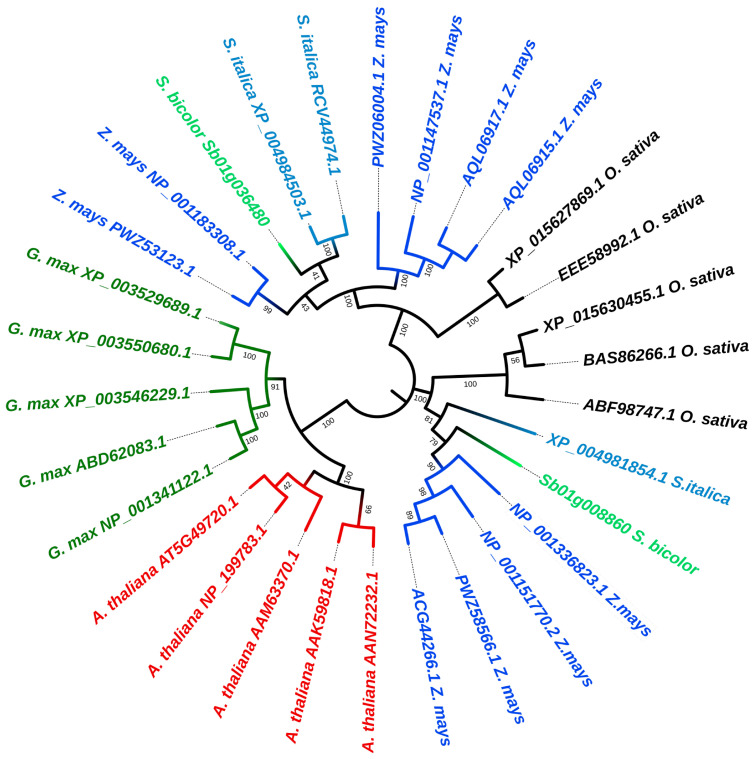
Phylogenetic analysis of KORRIGAN from different plant species. Maximum Likelihood phylogenetic tree of SbKOR1 in two dicots species and four types of grasses. SbKOR1 is highlighted. The tree placed SbKOR1 in the same clade as *Z. mays* endoglucanase 9, while its paralog was grouped with endoglucanase 10 protein from *Z. mays*. Phylogeny reconstruction was conducted with the Maximum Likelihood method, employing Jones-Taylor-Thornton (JTT) substitution model, and gramma distributed with invariant sites (G + I) method with 1000 bootstraps and visualized using the interactive tree of life (https://itol.embl.de/) accessed on 10 December 2022.

The complete protein sequence of SbKOR1 obtained from the Phytozome database was used to identify homologous proteins in two dicots species (*Glycine max* and *Arabidopsis thaliana*) and four monocot species (*Sorghum bicolor*, *Zea mays*, *Oryza sativa subsp. Japonica*, and *Setaria italica*) from the blast output (Figure 2). As evident from the phylogenetic tree, KOR protein from Arabidopsis and *G. max* are highly conserved among the species and formed a dicot specific sub-clade whereas in monocots the proteins have diverged further within each species. The tree showed SbKOR1 in the same clade as *Z. mays* endoglucanase 9, while its paralog Sb01g008860 (SbKOR2) was grouped with endoglucanase 10 protein from *Z. mays* (Figure 2).

### 2.3. Identification and Characterization of Additional thl Mutants

To identify other thick leaf mutants in sorghum, we screened a mini core collection of 256 EMS generated lines [31], which resulted in identifying two additional mutants, *thl2* and *thl3*. Mapping of the mutant loci of *thl2* and *thl3* revealed that the causal mutations are in the *Sb01g036480* (*SbKOR1*) locus, the same locus as *thl1* mutation (Supplementary Table S1 and Supplementary Figure S2). Causal mutations of *thl1* and *thl3* resulted in stop codons while causal mutation of *thl2* is a splice junction mutation. Premature codon results in production of a nonfunctional truncated protein. While splice site mutation might result in exon skipping or inclusion of introns altering the coding sequence of proteins resulting in splice variants [35].

### 2.4. Mutation in THL Resulted in Reduced Stem Crystalline Cellulose Content

**Figure 3:**
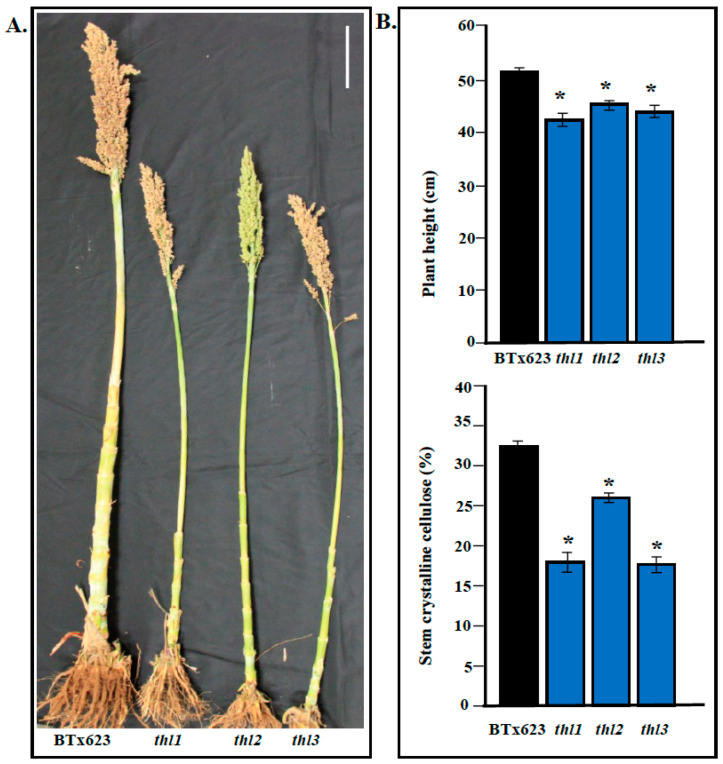
(**A**). Phenotype analysis of different *thl* mutants (*thl1*, *thl2*, *and thl3*). Scale bar: 10 cm. (**B**). *(***Upper panel**): Graph showing plant height differences between BTx623 and *thl* mutants (*thl1*, *thl2*, *thl3). (***B**). (**Lower panel**): Crystalline cellulose content analysis of BTx623 and *thl* mutants. * Represents significant difference.

Mapping data showed that the three *thl* mutant phenotypes are due to mutations in *KORRIGAN* gene. *KOR* gene is involved in cellulose biosynthesis, and mutation in Arabidopsis *KOR* results in reduced cellulose content [21,23]. To confirm the role of *THL* gene in cellulose biosynthesis, we estimated the cellulose content of *thl* mutants. Estimation of stem crystalline cellulose content at the mature stage showed a significant decrease (Figure 3) in cellulose content in *thl* mutants (*thl1*: 17.3%; *thl2*: 26.2% and *thl3*: 17.0%) compared to BTx623 (32.1%). Consistent with *thl1*, the two mutants (*thl2* and *thl3*) also showed reduction in plant height and crystalline cellulose content. Overall, the results obtained are consistent with the published Arabidopsis *kor* mutant, which showed that mutation in *KORRIGAN* results in the reduction of cellulose content [21,23].

### 2.5. Cell Elongation Is Compromised in thl Mutants

**Figure 4:**
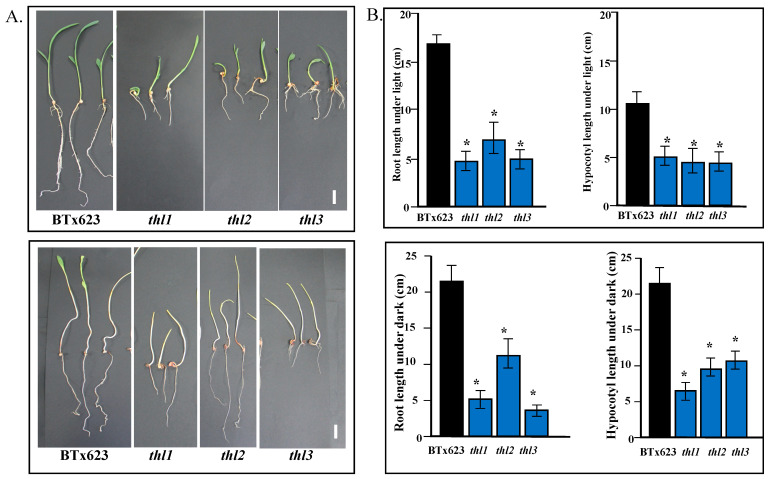
Light- and dark-grown seedlings of BTx623 and mutants *thl1*, *thl2*, and *thl3* were compared for root length and hypocotyl length. (**A**). One week to ten days old BTx623, *thl1*, *thl2*, and *thl3* seedlings grown under light conditions (**upper lane**). Scale bar: 1 cm. BTx623, *thl1*, *thl2*, *thl3* seedlings grown for 1 week to 10 days under dark conditions (**lower lane**). Scale bar: 1 cm. (**B**). Root length and hypocotyl length of BTx623 seedlings were compared with *thl1*, *thl2*, and *thl3* seedlings under light (**upper panel**). Root length and hypocotyl length of the seedlings under light was taller in BTx623 than in *thl1*, *thl2*, and *thl3*. Root length and hypocotyl length of BTx623 was compared with *thl1*, *thl2*, and *thl3* seedlings grown under dark (**lower panel**). Root length and hypocotyl length of the seedlings under dark was taller in BTx623 than in *thl1*, *thl2*, and *thl3*. * Represents significant difference.

Mutations in cell wall biosynthetic genes result in reduced cell elongation, and, as a result, the seedlings show reduced hypocotyls and root elongation [36]. Similarly, a mutation in Arabidopsis *KOR* showed reduced hypocotyl elongation when grown in dark conditions [21]. Analysis of light-grown and dark grown *thl* mutants (*thl1*, *thl2*, and *thl3*) and BTx623 showed that the root and hypocotyl elongation were significantly reduced in the mutants compared to BTx623 (Figure 4). The cross section of the 3-month-old leaves showed swollen cells in *thl1* mutant compared to BTx623 (Supplementary Figure S3). Overall, this data showed that the mutants might be compromised in cell elongation, cell wall biosynthesis, and, as a result, showed reduced root and hypocotyl elongation. 

### 2.6. Arabidopsis KOR Mutant (rsw2-1) Phenotype Is Restored by SbKOR1

**Figure 5:**
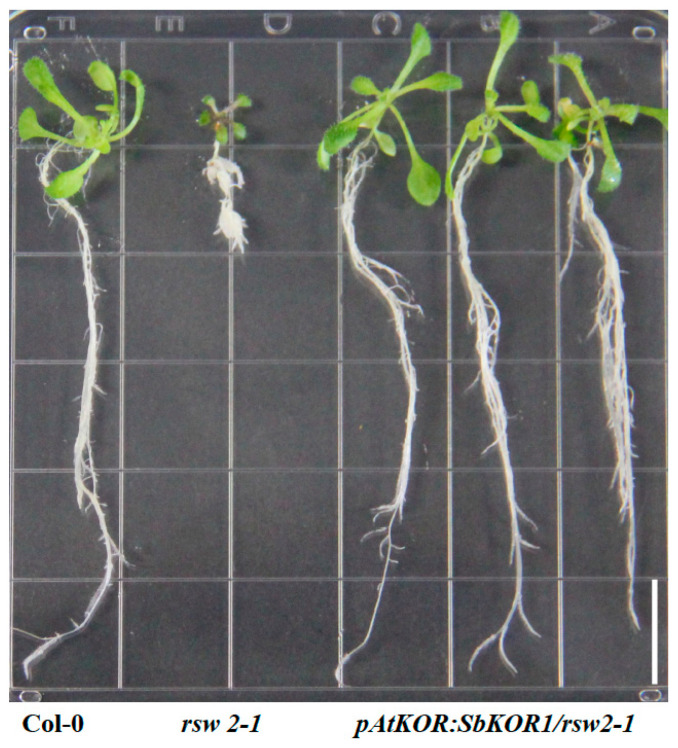
Rescue of phenotype. Arabidopsis complemented lines with *rsw2-1* mutant and Col-0. Scale bar 1 cm.

**Figure 6:**
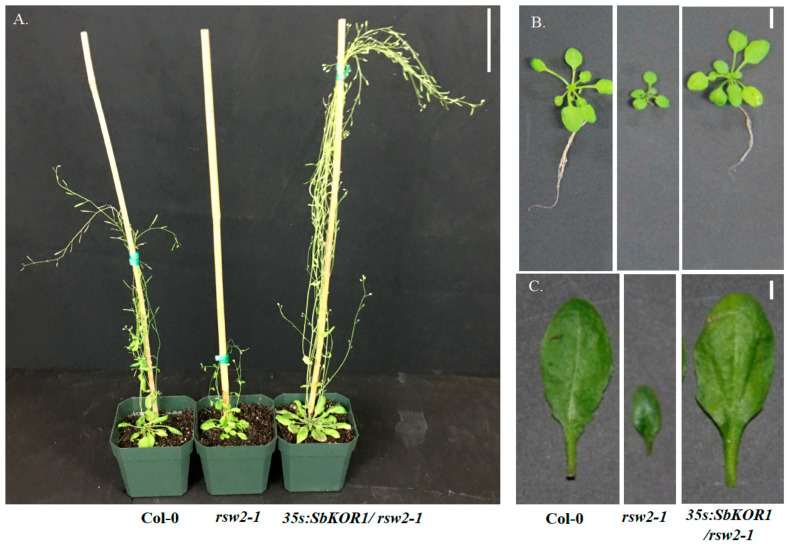
(**A**) Col-0 and *rsw2-1* mutant were compared with the transformed overexpression construct of sorghum, *SbKOR1* in *rsw 2-1* mutant of Arabidopsis (*35s:SbKOR1/rsw2-1*). Scale bar: 9 cm. (**B**) Whole plants of Col-0, *rsw2-1* mutant, and overexpressor (*35s:SbKOR1/rsw2-1*). Scale bar: 0.5 cm. (**C**) Leaf of Col-0, *rsw2-1* mutant, and Overexpressor (*35s:SbKOR1/rsw2-1*). Scale bar: 0.5 cm.

The BSAseq approach identified the causal mutations in *SbKOR1* as responsible for the *thick leaf* mutant phenotypes (*thl1*, *thl2*, *and thl3*). The three independent alleles of *thl* mutants provided strong evidence that (*SbKOR1*) the gene is responsible for the phenotype. To further demonstrate the function of this gene, we employed the Arabidopsis model system for functional complementation studies (Figure 5). The Arabidopsis model system was successfully used to characterize spruce (*Picea glauca*) *KORRIGAN* (*PgKOR*) [37], and we have successfully utilized the Arabidopsis model system to characterize soybean male sterile gene [38]. Arabidopsis *KOR* mutant (*rsw2-1*) was transformed with sorghum *KOR* (*SbKOR1*) gene using native Arabidopsis promoter (complementation) and constitutive CaMV35s promoter (overexpression) constructs (Supplementary Figure S4). Homozygous *rsw2-1* plants were transformed with the complementation and overexpression constructs independently. Homozygous T_2_ lines (complementation and overexpression) restored the *rsw2-1* phenotype at 31 °C (Figure 5 and Figure 6), confirming the fact that the *SbKOR1* is a functional endo-beta-1,4-endoglucanase in sorghum.

## 3. Discussion

Sorghum is a multipurpose crop with extensive applications in the food, fodder, biofuel, and fiber industries. The cell wall structure and composition of sorghum biomass play an important role in cell wall digestibility and are crucial for biofuel and animal feed industries. Cellulose synthesis and crystallinity are determined by proteins involved in the synthesis and processing. The *KORRIGAN* is an endo-beta-1,4-endoglucanase (EGases) that is known to untangle glucan chains to ensure synthesis of crystalline cellulose; however, the precise mechanism is not known. Unlike bacteria, plant EGases do not have a cellulose binding site, and therefore they are unable to hydrolyze crystalline cellulose [39,40]. The role of *KOR* in cellulose synthesis was reported in several plant species, such as Arabidopsis, poplar, rice, tomato, and white spruce [37,39,41,42,43]. Arabidopsis KORRIGAN is known to localize cells plate and regulate aberrant cell plates, incomplete cell walls, and multi-nucleated cells [2]. *KORRIGAN* orthologs have been found in other plant species, such as tomato [39] and poplar [43], and their role in cellulose synthesis was established. Functional characterization of *PtrKOR* in hybrid populus (*P. alba* × *P*. *grandidentata*) using RNA interference (RNAi) resulted in less cellulose content with higher crystallinity and increased xylan [43]. RNAi-mediated knockdown of *KOR* paralogs in *P. euramericana* resulted in reduced cellulose content and secondary cell wall thickness [44]. Overall studies of Arabidopsis and other species corroborated the role of the *KOR* gene in cellulose biosynthesis. Most of the studies related to *KORRIGAN* are aimed at altering the plant biomass characteristics for industrial applications [37,41,45]. Cell wall polysaccharides must be separated from lignin and then hydrolyzed with digestive enzymes to obtain fermentable glucan through saccharification for cellulosic ethanol production. Separation of cellulose from lignin and hydrolysis requires expensive treatments; hence, biomass with reduced cellulose crystallinity is important for reducing the processing costs. *KORRIGAN* is an ideal target to alter biomass traits due to its role in cellulose content and cellulose crystallinity. 

Studies by gel filtration analysis of Arabidopsis seedlings revealed that KORRIGAN is part of a CESA protein complex [24] and also shown to specifically interact with the cellulose synthase machinery [25]. Further, this association can be seen during the CSC trafficking from the Golgi stacks to the plasma membrane along the microtubules and at the plasma membrane using BiFC [24]. Based on the experimental data, it is hypothesized that KORRIGAN could have a role in the proof reading activity in untangling of aberrant glucan chain association of emerging glucans and allowing the production of highly crystalline cellulose [46] or determining the length of cellulose chain during cellulose synthesis [24] or cellulose microfibril release from CSC complex [4]. Considering strong bonds between the CSC and glucan chains, the protein could also cleave glucans to allow their incorporation into the cell wall while CSC is internalized. This could explain why *kor1-1* mutants show reduced GFP-CESA3 accumulation in microtubule-associated cellulose synthase compartments (compartments are used for CESA protein trafficking) being still tied to cellulose [24]. Thus, it was demonstrated that KOR1 not only associates with CSC complex but also helps in CSC intracellular trafficking.

Identification of three independent thick leaf mutants with similar phenotypes and causal mutation reaffirms role of *SbKOR1* in cellulose synthesis in sorghum. Phylogenetic analysis showed the presence of two *KORRIGAN* genes in sorghum; however, association of the three mutant alleles with *SbKOR1* shows its significance in cellulose synthesis in sorghum. It is also possible that the *SbKOR2* is expressed in different tissues or at different levels compared to *SbKOR1*. Therefore, it will be interesting to study the function of SbKOR2 protein and its role in cellulose biosynthesis and overall plant phenotype. Similar to *kor* mutant phenotype in Arabidopsis, our results confirmed the fact that mutation in *SbKOR1* gene results in the reduction of cellulose content [21,23]. It is interesting that the crystalline cellulose content of *thl2* is less reduced compared to *thl1* and *thl3*, which can be attributed to the fact that the *thl2* is not a stop codon mutation (splice junction mutant). It is possible that there are some functional transcripts produced in *thl2* mutant, resulting in only partial compromise in the *SbKOR1* function. Further, we also observed swollen cell phenotype in the leaf cross section of *thl1*. Defects in cell wall synthesis are known to cause radial cell swelling phenotypes due to weak wall that cannot withstand the internal cell turgor pressure. Reduced cellulose in Arabidopsis *rsw1* mutant resulted in radial swelling phenotype [47]. The *RSW1* locus codes for cellulose synthase catalytic subunit and mutation leads to radial swelling root phenotype under controlled temperatures. The *thl1* showed radial swelling phenotype in leaf cross section similar to *rsw1* root sections, indicating a weak cell wall in *thl* mutants due to reduced crystalline cellulose content (hence thick leaf phenotype). 

Studies showed that downregulation of *PtKOR1* and *PtKOR2* reduced overall plant growth with reduced stem internode length due to reduction in crystalline cellulose content. *SbKOR1* gene mutants (*thl1*, *thl2*, and *thl3*) showed similar phenotypes with concomitant reduction in crystalline cellulose content. Further, the functional complementation of Arabidopsis *rsw2-1* mutant by *SbKOR1* showed highly conserved function of *KOR* genes across plants in angiosperm clade. Gene orthologs of *KORRIGAN* are found in many plant species, yet its precise role in cellulose synthesis is still unclear. Discovering novel mutants from different species helps in improving the fundamental understanding of the nature of the gene function. A clear knowledge of the role of KOR protein function in cellulose biosynthesis could help us to alter the biomass for biofuel and bioproduct applications. Overall, the reported sorghum *thl* mutants serve as a valuable resource to study their role in cellulose biosynthesis and crystallinity as well as for utilization in biomass-based biofuel and animal feed industries.

## 4. Materials and Methods

### 4.1. Developing Mapping Population and Identification of thl Locus

Sorghum thick leaf mutants were isolated from the EMS (Ethyl Methane Sulfonate) mutant population of BTx623, as described previously [30,48]. The first generation of *thl* mutants was backcrossed with parental line BTx623 to remove background mutations. A mapping population for *thl* mutants was generated by crossing *thl1*, *thl2*, and *thl3*, with a male sterile line (*ms8*). Homozygous *thl* phenotypes were selected from the segregating population, and DNA was extracted from 40 homozygous mutant plants, pooled, and sequenced with the NextGen sequencing (https://www.bgi.com/us/home, accessed on 25 November 2022). The causal mutations of the *thl* mutants were analyzed with the BSAseq (Bulked segregant analysis) workflow on Cyverse (https://cyverse.org/, accessed on 25 November 2022) as described [34]. BSA seq is a genetic mapping technique that uses genomic sequences pool of a population of individuals segregating for the desired trait to identify and locate SNPs in the genome [26,49]. Results of the pipeline were visualized by downloading the total data from CyVerse data store. The mapped loci were confirmed by Sanger sequencing.

### 4.2. Plant Growth Conditions, Phenotyping, and Preparation of Samples for Estimation of Crystalline Cellulose Content

The three *thl* mutant lines *thl1*, *thl2*, and *thl3*, along with BTx623, were planted in the experimental field at USDA-ARS, Lubbock, Texas at 33°35′ N, longitude 101°53′ W with an altitude of 958 m made up of Amarillo fine sandy soil. Mature 3-month-old sorghum plants were collected in triplicates and phenotyped for various traits (Figure 1). The stem tissue was collected from the field from all *thl* mutants and BTx623 for the estimation of crystalline cellulose content. Another set of *thl* mutants along with BTx623 were grown in the green house for 12 days for seedling stage phenotyping. The greenhouse conditions were maintained at 28 °C/22 °C for 16 h a day and 8 h night cycle. The stems were separated from leaves, cut into smaller pieces, and air-dried for 24 h in the oven for 10 days at 49 °C. The dried biomass was ground into powder (2-mm particle size) using a 2-mm screen in a Wiley mill biomass grinder. Further, the samples were finely ground into homogenous powder using 6775 freezer/Mill^®^ using liquid nitrogen. Cellulose content of individual samples was estimated using Updegraff method [50] as described [51,52]. Briefly, the alcohol insoluble residue was treated with Updegraff reagent at 100 °C for 30 min followed by water and acetone washes. The resultant pellet was dried overnight at 37 °C and dissolved in 67 % sulphuric acid to estimate the crystalline cellulose content using Anthrone reagent [53]. The absorbance was measured at 620 nm using DTX880 multi-well plate reader (Beckman Coulter, Brea, CA, USA), and the crystalline cellulose content was measured using a standard curve derived from various concentrations of glucose. The cellulose content was averaged from three biological replicates for both *thl* mutants and BTx623 lines.

### 4.3. Seedling Growth Conditions and Phenotyping under Light and Dark Conditions

The mutant (*thl*) and control (BTx623) seeds were imbibed in water for 12 h in a 1.5 mL Eppendorf tube and germinated on a moist paper towel both under light and dark conditions at room temperature growth chambers (Percival^TM^, Perry, IA, USA). Dark conditions were maintained by wrapping the seeds in moist paper towels in an aluminum foil to block the light while the light experiment seedlings were exposed to light conditions at room temperature in growth chambers (Percival^TM^, Perry, IA, USA). After 12 days of incubation, seedling morphology was evaluated by root and hypocotyl length. Images of seedlings were taken separately for light and dark conditions for BTx623, *thl1*, *thl2*, and *thl3* mutants using a Canon EOS60D camera, and obtained images were analyzed using ImageJ (1.5.3) software.

### 4.4. Histochemical Analysis of Leaf Cross Sections

Three biological replicates of 3-month-old BTx623 and *thl1* mutant plants were used for cross-section analysis. Fresh leaf and stem samples were cut and immediately transferred into vials containing the fixative solution (formaldehyde:acetic acid:ethanol in a 2:1:10 ratio). The fixed sections were vacuum infiltrated in a desiccator for an hour to clear all the air bubbles from the tissue and stored at 4 °C. Later, the fixed tissues were dehydrated using a series of ethanol and xylene exchanges. A series of paraffin wax exchanges were conducted to embed tissues into paraffin blocks. The fixed tissues in paraffin molds were sectioned with the aid of a microtome to obtain sections of 5 μm thickness. The sections were moved onto Paraplast plus slides and dried overnight at 37 °C, followed by rehydration. After rehydration, the slides were stained using 0.05% Toluidine blue (Sigma Aldrich, St. Louis, MO, USA), and images were taken using an Olympus DP80 camera connected to cell scan software. 

### 4.5. Arabidopsis Transformation

Arabidopsis plants were grown in soil or on ½MS (Murashige Skoog) medium with 2% sucrose medium in plant growth chambers (Percival^TM^) maintained under long-day conditions (22 °C -day/20 °C -night with 16 h day/8 h night cycle) supplied with a light intensity of 12,200 lux (fluorescence) units. For screening the transgenic lines, the seeds were placed on ½MS medium with selection agent, Hygromycin at 50 μg/mL concentration and monitored for phenotypic characters of the transgenic lines. Only transgenic plants that survived on the selection media were transferred onto the soil in pots (Sun grow mix 900 soils) for further analysis. The seedlings were grown on vertically oriented plates with MS media for root phenotype analysis. The temperature sensitive mutant *rsw 2-1* (At5g49720; Cs-6555) with Columbia 0 (Col-0) background was grown in soil under long-day conditions at 21 °C. The germplasm information of the *Arabidopsis thaliana* T-DNA insertion mutants is available at the *Arabidopsis* Information Resource Center (TAIR, www.Arabidopsis.org) accessed on 15 March 2018.

### 4.6. RNA Extraction from Plant Tissue, cDNA Synthesis, and Gene Amplification

Total RNA was extracted from 100 mg sorghum wild-type BTx623 plant samples using Spectrum^TM^ Plant Total RNA kit (Sigma-Aldrich, St. Louis, MO, USA). The extracted RNA samples were subjected to on-Column *DNase I* (Sigma-Aldrich, USA) digestion to remove genomic DNA contamination. The concentration of the extracted RNA was measured using a UV spectrophotometer (BioSpectrophotometer Ò Kinetic, Eppendorf, Hamburg, Germany) at 260 nm. The quality of the total RNA extracted was analyzed on 1% agarose gels. A total RNA of 1 μg was used for first strand cDNA synthesis following the manufacturer instructions of Invitrogen superscript 2Ò reverse transcriptase kit (Invitrogen, Carlsbad, CA, USA). Primers for *SbKOR1* were designed using primer 3 software with appropriate parameters for optimal primer designing [54]. The coding region of the *SbKOR1* gene was used to design forward and reverse primers (Supplementary Table S1). The melting temperatures were set above 60 °C, with no 3′ or 5′ end complementarity between primers. The 1.855 kb DNA fragment was amplified using *SbKOR1* forward and reverse primers (Supplement Table S1).

### 4.7. Identification of Sorghum Homologs

The Glycosyl hydrolase family 9 genes were identified using protein sequences of *Arabidopsis thaliana* endo-1, 4-beta-D-glucanase (*AtKOR1*). The Arabidopsis data were retrieved from Tair10 (The *Arabidopsis* Information Resource) to perform a genome-wide similarity search with sorghum genomes. The similarity test was performed using BLASTN and BLASTP for coding and protein sequences using an e-value cut off 10^−10^. 

### 4.8. Phylogenetic Tree Construction and Sequence Alignment 

*SbKOR1* homologs from *Glycine max*, *Arabidopsis thaliana*, *Sorghum bicolor*, *Zea mays*, *Oryza sativa* subsp. Japonica, and *Setaria italica* were obtained from the NCBI database (https://www.ncbi.nlm.nih.gov/) accessed on 24 October 2022 using BLASTP. Phylogenetic analysis identified the presence of two *KORRIGAN* genes in sorghum: *Sb01g036480 (SbKOR1)* and *Sb01g008860 (SbKOR2)*. The alignment was performed using CLUSTALW embedded in MEGA11 with the default setting. Phylogeny reconstruction was conducted with the Maximum Likelihood method, employing the Jones-Taylor-Thornton (JTT) substitution model, and gamma distributed with invariant sites (G + I) method with 1000 bootstraps and visualized using the interactive tree of life (https://itol.embl.de/) accessed on 10 December 2022 (Figure 2). Twenty-nine homologous proteins were identified from these six generas, which had more than 70% sequence similarity and query coverage. 

### 4.9. Cloning, Overexpression, and Complementation of SbKOR1

For overexpression of sorghum *SbKOR1* gene in Arabidopsis (Col-0), the predicted 1863 bp coding sequence (CDS) of *SbKOR1* gene was amplified from sorghum wild type BTx623 cDNA library using NEB Next high-fidelity 2X PCR Master Mix (New England Biolabs, Ipswich, MA, USA). The PCR product was then digested with *AscI* and *PacI* (New England Biolabs, Ipswich, MA, USA) restriction enzymes and sub-cloned into the *AscI* and *PacI* digested pMDC32 binary vector. For complementation, the predicted 3 kb of *AtKOR* native promoter was amplified from *Arabidopsis thaliana* (Col-0) using NEB Next high-fidelity 2X PCR Master Mix (New England Biolabs, USA). The PCR product was then digested with *SbfI* and *AscI* (New England Biolabs, USA) and sub-cloned into the *SbfI* and *AscI* digested pMDC32 binary vector containing *SbKOR1* genes. The digested product of pMDC32 binary vector with 1863 bp CDS of *SbKOR1* gene was ligated with digested KOR native promoter from *Arabidopsis* (Col-0) 3kb. The over-expression construct was confirmed by Sanger sequencing (GenewizTM, South Plainfield, NJ, USA). The gene fragments of the overexpression construct were PCR amplified using (Supplementary Table S1) specific primers, gel purified, and sequenced. The sequencing results were analyzed by using BLASTN.

### 4.10. Plant Growth and Agrobacterium Mediated Plant Transformation

*Arabidopsis KORRIGAN* mutant line, *rsw2-1* (*At5g49720*), and *Arabidopsis* seeds were sterilized in 20% Clorox solution for 20 min by continuous shaking on a shaker followed by washes with sterile water. Seeds were placed on a ½MS selection medium with 2% sucrose containing 50 μg/mL concentration of Hygromycin [55]. The seedlings grown on the ½MS media were transplanted into soil and grown in a growth chamber. The flowering plants were transformed using floral dip transformation according to the published protocol [56]. The overexpression construct and complementation constructs containing *SbKOR1* were transformed independently into *Arabidopsis rsw2-1* (At5g49720) mutant plants. Transgenic events were selected on media with Hygromycin (50μg/mL) and independent events were transferred onto soil. The transgenic (T_0_) seeds of the plants that survived on the selection media were transplanted in soil after 7 days, and T_1_ seeds were collected separately from individual transgenic events. The T_1_ plants were germinated on selection media and transplanted in the soil. Homozygous T_2_ seeds of overexpression, complemented lines along with *rsw2-1* mutant, and Col-0 were used for phenotype analysis. Each plant represents an individual line, and the seeds were collected separately. Temperature-sensitive *rsw2*-1 mutant transgenics (overexpression and complementary lines) were kept under two distinct temperatures, 21 °C and 31 °C, for phenotypic analysis. The plants were grown at 22 °C in dark for 3 days and maintained at 31 °C continuously in 16 h day/8 h night cycle to test the root morphology change in the *rsw2-1* mutant [29].

### 4.11. Statistical Analysis

A non-parametric test of independent two-group Mann–Whitney U Test (*t*-test) has been performed for significant difference in medians between the two groups for crystalline cellulose content between BTx623 and mutants (BTx623 vs. *thl1*, BTx623 vs. *thl2*, and BTx623 vs. *thl3*). The *p*-values of these groups were compared at a 5 % level of significance (*p*-value (α) = 0.05). The Kruskal–Wallis test was employed to assess the significant differences in root and hypocotyl length of BTx623, *thl1*, *thl2*, and *thl3* in both light and dark conditions.

## Data Availability

Not applicable.

## Supplementary Materials

The following supporting information can be downloaded at: https://www.mdpi.com/article/10.3390/plants11243531/s1, Supplementary Figure S1: Gene structure of *AtKOR* (AT5G49720) and *SbKOR1* (Sb01g036480) with 6 exons and 5 introns; Supplementary Figure S2: Alignment of AtKOR1 and Sb01G036480 (SbKOR1) proteins; Supplementary Figure S3: Leaf cross section of BTx623 and *thl1*; Supplementary Figure S4. Vector construction maps. Supplementary Table S1: Identification of mutant locus using BSAseq; Supplementary Table S2: Primers used for experiments and sequence.
