# Supplementary material for: Mutation in the Endo-β-1,4-glucanase (KORRIGAN) Is Responsible for Thick Leaf Phenotype in Sorghum
Source: Plants (Basel). 2022 Dec 15;11(24):3531. doi: 10.3390/plants11243531 (PMC9780866; doi:10.3390/plants11243531)
Supplement: Supplementary file 1 [file plants-11-03531-s001.zip › plants-2090475-supplementary.pdf]

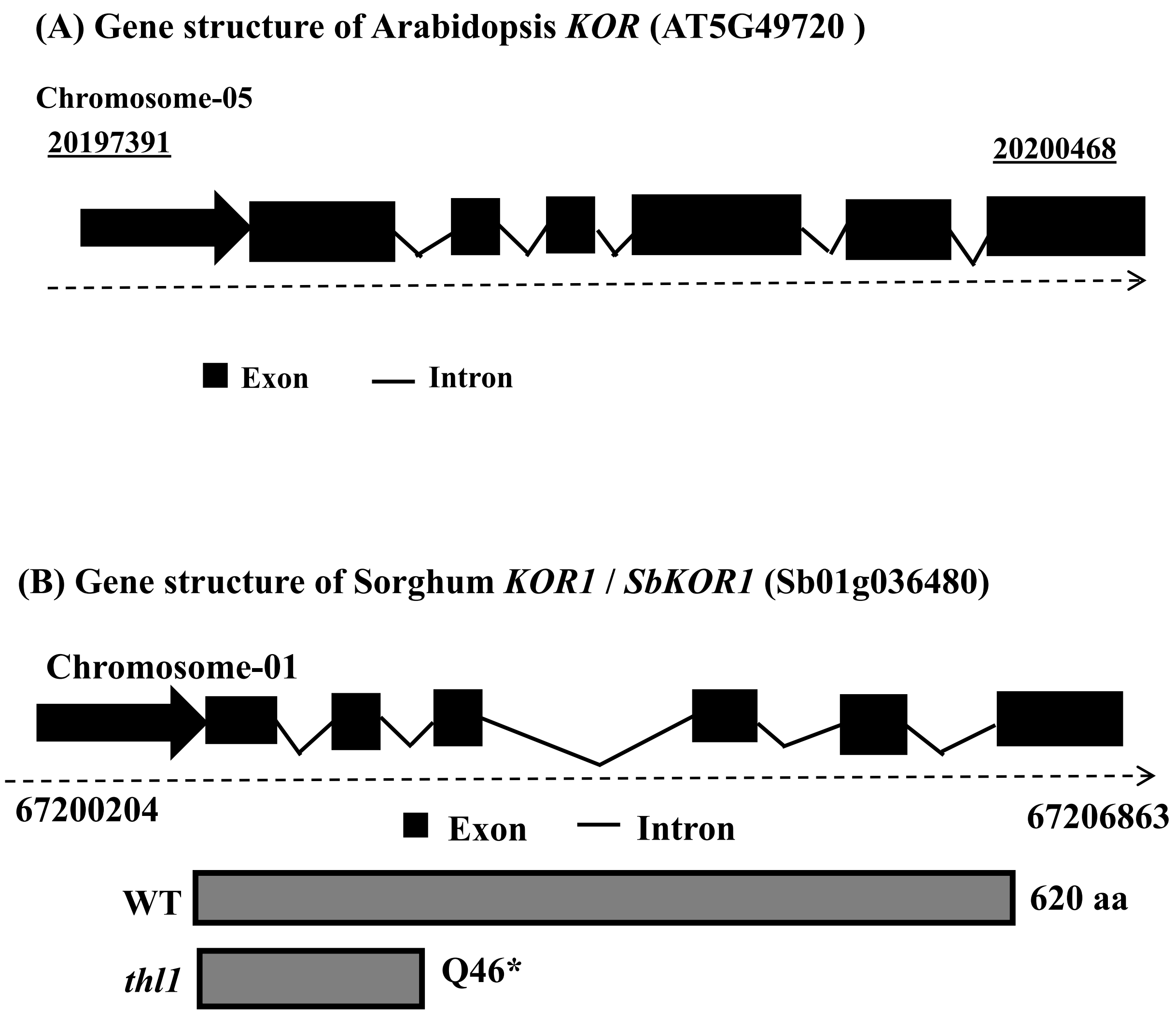

Supplementary Figure S1: Gene structure of *AtKOR* (AT5G49720) and *SbKOR1* (Sb01g036480) with 6 exons and 5 introns.

```
CLUSTAL O(1.2.4) multiple sequence alignment


ATKOR           --MYGRDPWGGPLEINTADSATDDDRSRNLNDLDRAALSRPLDETQQSWLLGP---TEQK    55
SB01G036480     MSMYGRDPWGGPLEICHPDSATDDDRSRNL-DIDRGAMSRTLDETQQSWLLAGPGDQARK    59
                  *************   ************ *:**.*:** **********.      :*

ATKOR           KKKYVDLGCIIVSRKIFVWTVGTLVAAALLAGFITLIVKTVPRHHPKTPPPDNYTIALHK    115
SB01G036480     KKKYVDIGCLVVSRKLFVWTLGVLLAAAVFAGVVAGIAKAIPRRHRPPPPPDDYTVALHK    119
                ******:**::****:****:*.*:***::**.:: *.*::**:*   ****:**:****

ATKOR           ALKFFNAQKSGKLPKHNNVSWRGNSGLQDGKGETGSFYKDLVGGYYDAGDAIKFNFPMAY    175
SB01G036480     ALMFFNAQRSGKLPKHNNIPWRGNSCMKDGLSDP-AVRRSLVGGYYDAGGAVKFNFPAAF    178
                ** *****:*********: *****  ::**  .:   :.  :.*********.*:***** *:

ATKOR           AMTMLSWSVIEYSAKYEAAGELTHVKELIKWGTDYFLKTFNSTADSIDDLVSQVGSGNTD    235
SB01G036480     SMTLLSWSVIEYSAKYDAVGELGHVRDIIKWGSDYFLKTFNSTADSIDRVIAQVGSAAMS    238
                :**:************:*.*** **:::****:*************** :::****.   .

ATKOR           DGNTDPNDHYCWMRPEDMDYKRPVTTCNGGCSDLAAEMAAALASASIVFKDNKEYSKKLV    295
SB01G036480     PGSTQPNDQYCWMRPEDIDYPRPVVECH-ACSDLAAEMAAALAAASIVFKDNKAYSQKLV    297
                 *.*:***:********:** ***. *: .*************:********* **:***

ATKOR           HGAKVVYQFGRTRRGRYSAGTAESSKFYNSSMYWDEFIWGGAWMYYATGNVTYLNLITQP    355
SB01G036480     HGATTLFQFARERRGRYSAGGSDATKFYNSTSYWDEFVWGSSWMYLATGNSSYLTLATHP    357
                ***..::**.* ******** ::::*****: *****:**.:*** **** :**.* *:*

ATKOR           TMAKHAGAFWGGPYYGVFSWDNKLAGAQLLLSRLRLFLSPGYPYEEILRTFHNQTSIVMC    415
SB01G036480     KLAKHAGAFWGGPDYGVFSWDNKLTGAQVLLSRLRLFLSPGYPYEEMLRAFHNQTSIIMC    417
                .:********** **********:***:****************:**:*******:**

ATKOR           SYLPIFNKFNRTNGGLIELNHGAPQPLQYSVNAAFLATLYSDYLDAADTPGWYCGPNFYS    475
SB01G036480     SYLPIFKSFNRTRGGLIQLNHGKPQPLQYVVNAAFLASVFSDYLEAADTPGWYCGPHFYS    477
                ******:.****.****:**** ****** *******:::****:***********:***

ATKOR           TSVLRDFARSQIDYILGKNPRKMSYVVGFGTKYPRHVHHRGASIPKNKVKYNCKGGWKWR    535
SB01G036480     IEVLRSFARTQIEYILGKNPLKMSYVVGFGNHYPKHVHHRGASIPKNGVHYGCKGGWKWR    537
                 .***.***:**:******* *********.:**:************ *:*.*******

ATKOR           DSKKPNPNTIEGAMVAGPDKRDGYRDVRMNYNYTEPTLAGNAGLVAALVALSGEEEATGK    595
SB01G036480     DTKKPNPNIIVGAMVAGPDRHDRFKDARKNYNYTEATLVGNAGLVAALVALSGEGH---G    594
                *:****** * ********::* ::*.* ****** **.************** .

ATKOR           IDKNTIFSAVPPLFPTPPPPPAPWKP      621
SB01G036480     VDKNAMFSAVPPMFPSPPPPPAPWKP      620
                :***::******:**:**********
```

```
CLUSTAL O(1.2.4) multiple sequence alignment


ATKOR           --MYGRDPWGGPLEINTADSATDDDRSRNLNDLDRAALSRPLDETQQSWLLGPTEQKKKK 58
thl2            MSMYGRDPWGGPLEICHPDSATDDDRSRNL-DIDRGAMSRTLDETQ-------------- 45
                  *************   ************ *:**.*:** *****

ATKOR           YVDLGCIIVSRKIFVWTVGTLVAAALLAGFITLIVKTVPRHHPKTPPPDNYTIALHKALK 118
thl2            ------------------------------------------------------------ 45

ATKOR           FFNAQKSGKLPKHNNVSWRGNSGLQDGKGETGSFYKDLVGGYYDAGDAIKFNFPMAYAMT 178
thl2            ------------------------------------------------------------ 45

ATKOR           MLSWSVIEYSAKYEAAGELTHVKELIKWGTDYFLKTFNSTADSIDDLVSQVGSGNTDDGN 238
thl2            ------------------------------------------------------------ 45

ATKOR           TDPNDHYCWMRPEDMDYKRPVTTCNGGCSDLAAEMAAALASASIVFKDNKEYSKKLVHGA 298
thl2            ------------------------------------------------------------ 45

ATKOR           KVVYQFGRTRRGRYSAGTAESSKFYNSSMYWDEFIWGGAWMYYATGNVTYLNLITQPTMA 358
thl2            ------------------------------------------------------------ 45

ATKOR           KHAGAFWGGPYYGVFSWDNKLAGAQLLLSRLRLFLSPGYPYEEILRTFHNQTSIVMCSYL 418
thl2            ------------------------------------------------------------ 45

ATKOR           PIFNKFNRTNGGLIELNHGAPQPLQYSVNAAFLATLYSDYLDAADTPGWYCGPNFYSTSV 478
thl2            ------------------------------------------------------------ 45

ATKOR           LRDFARSQIDYILGKNPRKMSYVVGFGTKYPRHVHHRGASIPKNKVKYNCKGGWKWRDSK 538
thl2            ------------------------------------------------------------ 45

ATKOR           KPNPNTIEGAMVAGPDKRDGYRDVRMNYNYTEPTLAGNAGLVAALVALSGEEEATGKIDK 598
thl2            ------------------------------------------------------------ 45

ATKOR           NTIFSAVPPLFPTPPPPPAPWKP      621
thl2            -----------------------      45
```

Supplementary Figure S2: Alignment of AtKOR1 and Sb01G036480 (SbKOR1) proteins.

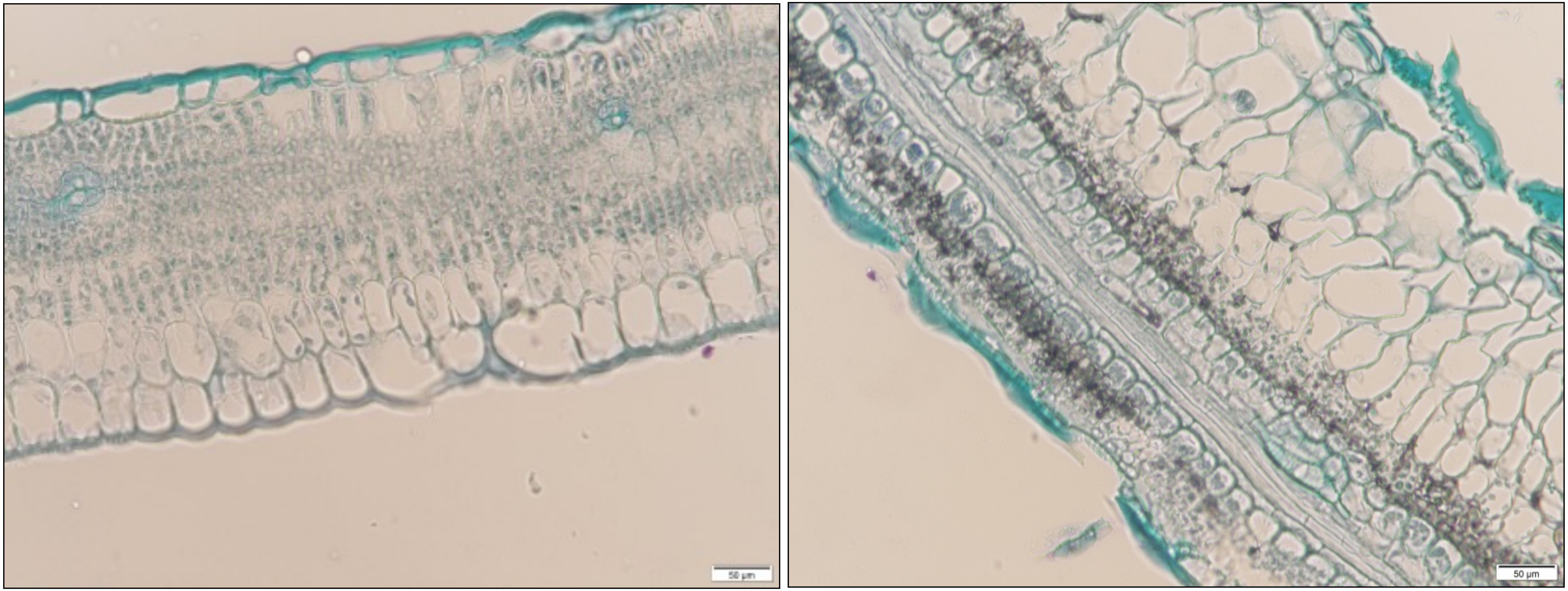

BTx623 ***thl1***

Supplementary Figure S3. Leaf cross section of BTx623 and *thl1* (Scale bar: 50 µm)

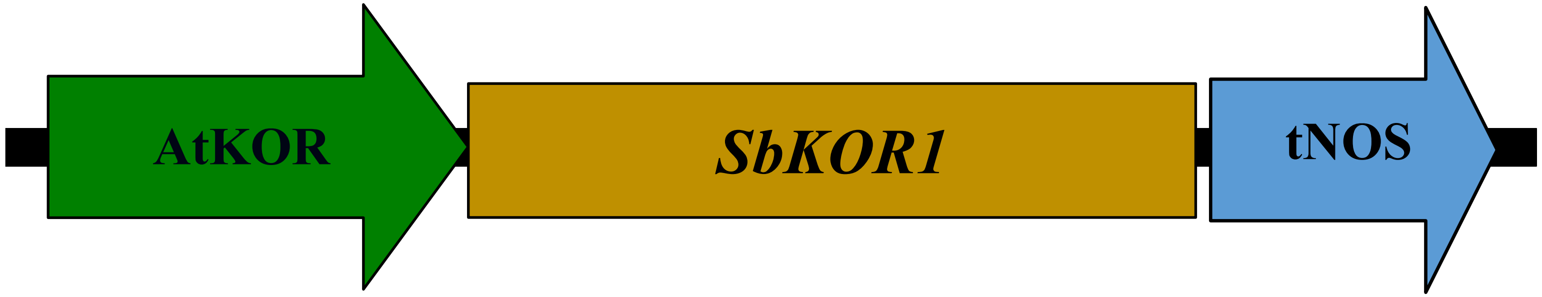

Complementation construct with native promoter

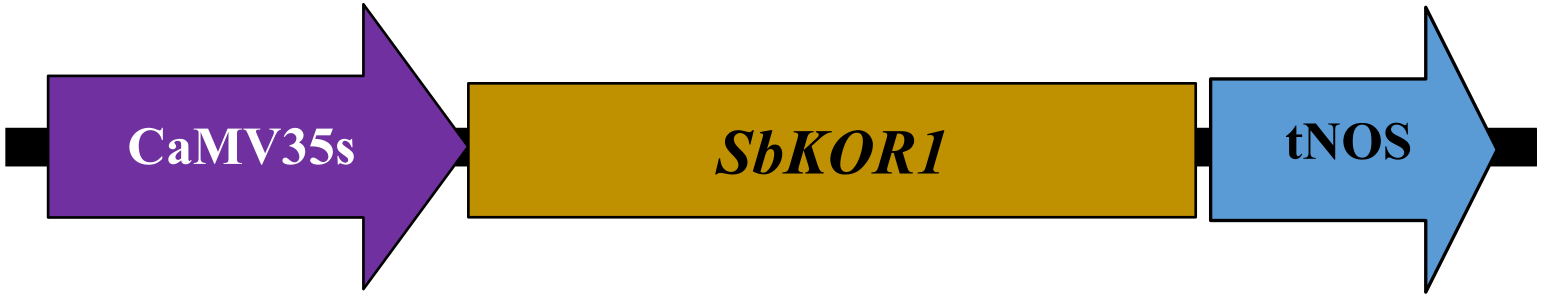

Over expression construct

Supplementary Figure S4. Vector construction maps.

Supplementary Table S1: Identification of mutant locus using BSAseq.

**Mutant**	**Mutation**	**Gene ID (Sorghum)**	**Gene ID (Arabidopsis)**	**Gene function**	**Nature of the mutation**
thl1	16QK6341 to 6352	Sb01g036480	AT5G49720.1	glycosyl hydrolase 9A1	Chr01:60010687;Q46*;STOP_GAINED;homo
thl2	16QK6353 to 6364	Sb01g036480	AT5G49720.1	glycosyl hydrolase 9A1	Chr01:60010937;SPLICE_SITE_DONOR;;homo
thl3	16QK6365 to 6376	Sb01g036480	AT5G49720.1	glycosyl hydrolase 9A1	Chr01:60010784;W78*;STOP_GAINED;homo

Supplementary Table S2: Primers used for experiments and sequence

Primer number	Primer description	Primer sequence
1465	1465_ACoa_Over_ ASCI F	gggcGGCGCGCCATGTTAGAAACTAACGAGCAAAAG
1466	1466_ACoa_Over_ PacI R	taaaAATTAATTTCAGACACACCGGTGTGGAGAGTC
1520	1520_SbKOR1_Asc1F	gcttGGCGCGCCATGAGCATGTACGGGAGGGA
1521	1521_SbKOR1_Pac1F	ggccTTAATTAATTATGGTTTCCATGGGGCCG
1522	1522_At_KOR1prom_SBF1	gcttCCTGCAGG TTTTAGAGCTTGTAAGATCCTACAAC
1523	1523_At_KOR1prom_ASC1	gcttGGCGCGCC ATATTTTTGGCCAACTATGATTGAGCG
1590	1590_Atkori2-1_F_ASCI	gcttCGATGCTTTCAAGGCCAAAGAAC
1591	1591_Atkori2-1_R_PacI	ggccCGTCATCATCCTAAGACTCCGCC
2274	2274_Kor_genotyping_F	GTTGTTGGATGGATCATTCAAGG
2275	2275_Kor_genotyping_R	CAAAGACTCTTTGAGAGATCTGG
2276	2276_rsw_genotyping_F	GCTGGGACAACAAGCTTGCTGG
2277	2277_rsw_genotyping_R	CCATCCAGGAGTATCAGCAGC
2303	2303_SbKOR1_Asc1-FP	aaggttGGCGCGCCgATGAGCATGTACGGGAGGGACCCG
2304	2304_SbKOR1_Pac1-RP	ggccggTTAATTAATTATGGTTTCCATGGGGCCGGTGGC
2305	2305_At_KOR1prom_SBF1-FP	gttccCCTGCAGGcgatgctttcaaggccaaagaac
2306	2306_At_KOR1prom_ASC1-RP	caaggccGGCGCGCCgatgatgctctctgataaagc
708	708_qPCRSbKOR-F	GATGAGACCAGAGGACATCGACT
709	709_qPCRSbKOR-R	CTTGCAAACTGGAAGAGAGTGGT
